# Civil servants' demand for social health insurance in Northwest Ethiopia

**DOI:** 10.1186/s13690-018-0297-x

**Published:** 2018-09-13

**Authors:** Sahilu Yeshiwas, Mengistu Kiflie, Atinkut Alamirrew Zeleke, Mihiretu Kebede

**Affiliations:** 1 Institute of Public Health, Department of Health Informatics, University of Gondar, Gondar, Ethiopia; 2 Federal Ministry of Health, Addis Ababa, Ethiopia; 3 Leibniz Institute for Prevention Research and Epidemiology – BIPS, Department Prevention and Evaluation, Unit Applied Health Intervention Research, Achterstraße 30, D-28359 Bremen, Germany

**Keywords:** Demand, Social health insurance, Health insurance, Civil servants

## Abstract

**Background:**

Absence of reliable health insurance schemes is a key challenge to meet the universal health coverage target of the Sustainable Development Goals (SDGs). Ethiopian health system is characterized by under financing, low protection mechanisms for the poor, and lack of mechanisms of risk pooling and cost sharing. Ethiopia is implementing social health insurance (SHI) scheme to reduce out of pocket payment (OOP) and improve access and use of healthcare. This study aimed to determine the demand for SHI among civil servants and associated factors in Northwest Ethiopia.

**Methods:**

An institution-based cross-sectional study was conducted in Bahir Dar city from 557 randomly selected civil servants using structured and self-administered questionaire. The questionnaire included questions measuring demand for SHI and demographic, socio-economic, healthcare related and personal and behavioral factors. Data were first entered in Epi-Info version 7.0 and transferred to SPSS version 20 for analysis. Descriptive statistics, bivariate and multivariable logistic regression analysis were performed.

**Results:**

From the total calculated sample size of 557, 488 respondents returned the questionnaire giving a response rate of 88%. Nearly three-fourth of the respondents, 355 (72.7%), reported their need to be enrolled in a SHI scheme. Two-third of the respondents 325 (66.6%) were willing to pay for their enrollment. Overall, three hundred and two (61.9%) were demanding SHI. Having good awareness about health insurance [AOR = 4.39, 95% CI = (1.82–12.89)] and trust on a health insurance agency [AOR = 3.0, 95% CI = (1.57–5.72)], were significantly associated with the demand for SHI among civil servants.

**Conclusion:**

The demand for SHI among civil servants were higher. The awareness towards SHI and trust on the SHI agency were significantly associated with demand for SHI. As Ethiopia aspires to insure all employees of the formal sector, and improving the awareness of civil servants about SHI and the agency providing the service could improve demand for SHI. Further research is important on healthcare organizational and professional readiness to handle the upcoming insurance driven quality health service need and health seeking behavioral change.

**Electronic supplementary material:**

The online version of this article (10.1186/s13690-018-0297-x) contains supplementary material, which is available to authorized users.

## Background

At least half of the world population lacks essential health services [1]. Universal health coverage through affordable healthcare is one of the main targets of sustainable development goals (SDGs) to be met by the end of 2013 [1–3]. A major barrier to this target is the frequently unaffordability of the cost of healthcare to the people, and that the need for such care is often uncertain [4–7]. This has affected an estimated 1.3 billion people around the globe to lack access to healthcare. A survey on 89 countries suggested that an estimated 150 million people suffer financial catastrophes due to out-of-pocket payment (OOP) for health service [8]. In 2017, the WHO reported that an estimated 800 million people, 12% of the world population, paid at least 10% of their household budget for healthcare [1]. As a result, more than 100 million people are pushed to poverty due to catastrophic health spending [9]. Catastrophic spending for healthcare, defined as “*paying more than 40% of household income directly on health care after basic needs have been met”*, occurs in countries at all income levels, but, it is greatest in those that rely most on direct payments to raise funds for healthcare [10]. In most low income countries where government expenditure on health is low, 85% of the cost for healthcare is covered by out of pocket payment (OOP) [11].

The removal of user fees aiming to reduce financial barriers [12] and health financing through risk pooling mechanisms [10, 13] and giving special attention to the poor [14] promote universal health coverage and equity. The implication of health financing through risk pooling mechanisms is that the healthy will pay for some or all of the health care services used by the sick and if this mechanism advances, the wealthier will pay for the services used by the poor [15]. Health insurance reduces catastrophic health spending, improves access and use of health care which ultimately improves health outcomes [16, 17].

The Ethiopian health system is characterized by extreme under financing, low protection mechanisms for the poor, and lack ways of risk pooling and cost sharing; all of which result in inequality in access to healthcare. Data from OOP health expenditure trend reported by World Bank showed that Ethiopia remained one of the highest (78%) from 1995 to 2014 with no improvement between these years. For many households in Ethiopia, a small OOP payments can result in financial catastrophes [18]. A steady drip of medical bills force people with chronic diseases or disabilities into poverty [19, 20].

Since the Ethiopian government parliament ratified health insurance in 2011, the government struggles to start compulsory social health insurance and community based health insurance for the formal and informal sectors respectively. The implementation of all forms of health insurance systems in Ethiopia are at the earliest stages of development. In general, health insurance has been nearly non-existent in Ethiopia [21]. Recently, the Ethiopian government has planned to implement Social Health Insurance (SHI) among employees of the formal sector. This health insurance is planned to provide health insurance for employees of the formal sector and their families. Active employees will have to pay a monthly premiums of 3% while pensioners are required to pay 1 % of their monthly salary [22, 23]. However, little is known about the demand for social health insurance. In general terms, whether an individual demand for SHI and is willing to pay for it depends on the perceived difference between the level of expected utility with insurance and expected utility without insurance [24, 25]. As to the knowledge of the investigators, there is no study in Ethiopia about the demand for social health insurance. Sound understanding of factors associated with demand for SHI among civil servants is important before the promotion and expansion of SHI. Therefore, this study aimed to determine demand for social health insurance and identify associated factors among civil servants of Northwest Ethiopia.

## Methods

### Study setting

Institution-based cross-sectional study was carried out in Bahir Dar city administration, Ethiopia. Bahir Dar is one of the biggest cities in Ethiopia and it is the capital of Amhara National Regional State, located 565 Km away from Addis Ababa, the capital of Ethiopia. An estimated 220,344 inhabitants live in Bahir Dar city. During the study period, the town had 3225 civil servants working in 27 government sectors.

One public referral hospital, ten public health center, three higher and seven special higher private clinics, and two private hospitals are available in the town. The government health institutions are financed by the government, donors and out of pocket expenditure collected from patients during the time they receive healthcare service.

#### Study population and sampling strategy

Civil servants who live in Bahir Dar and work in one of government institutions in Bahir Dar town during the study period were randomly selected. A single population proportion formula was used to calculate the sample size using Open Epi software. We assumed proportion of civil servants demand for SHI as 50, 95% confidence interval, 4% absolute precision/margin of error, 10% non-response rate and a total population of 3225 civil servants. The total sample size was calculated to be 557. The sample was drawn proportionally from each of the 27 government sectors. These government sectors had a mean total population of 120 employees (SD = 280) (Additional file 1).

All government institutions available in Bahir Dar town were included in the study. Samples were allocated proportionally based on the size of civil servants in each government sector. The respondents in each government institutions were selected randomly by a computer generated random number using the payrolls register as a sampling frame.

### Variables of the study

Demand for SHI (dichotomized as “Demanding”, “Not demanding”) was the dependent variable of the study. Independent variables such as socio-demographic and socio-economic variables including age, sex, marital status, educational status, religion, number of dependent children, family size, total number of dependents, monthly income, job, work experience, spouse employment status, spouse education status, and spouse job were collected. Healthcare related variables such as physical access to health care, self-reported history of illness in the last 12 months, self-reported OOP expenditure, perceived satisfaction with the quality of healthcare services(satisfied/not satisfied), duration of illness, last 12 months hospitalization history (admitted/not admitted), duration of hospitalization, self-reported evaluation of the current healthcare service cost payment system. In addition, personal behavioral factors including awareness on the objectives, components, and benefit packages of SHI and attitude on health insurance were included. Physical access to healthcare was measured by a single question by asking how long it takes to reach the nearest health facility. Perceived satisfaction with the quality of healthcare services (satisfied/not satisfied) and self-reported evaluation of the current healthcare service cost payment system were measured each by a single question.

#### Operational definitions

##### Demand

The need to enroll in SHI and the willingness to pay for it. Therefore, a respondent was considered as “demanding SHI” when the answers for both questions measuring need and willingness to pay for SHI were ‘yes’, else it is considered as “not demanding for SHI”.

##### Awareness of SHI

measured by responses of 13 multiple choice questions. These questions include items measuring respondents’ level of basic knowledge about benefits, funding sources and packages of social health insurance. Detail items of the questionnaire can be accessed in Additional file 2. Those civil servants who score above the median value to the questions asked about awareness of SHI were considered as having “good awareness”, else “poor”.

##### Attitude

measured by asking 4 Likert scale questions (1 = strongly agree, 2 = agree, 3 = Uncertain, 4 = Disagree, 5 = strongly disagree). Because the total attitude score was not normally distributed, median was preferred instead of mean. Therefore, civil servants who score above the median value to the questions asked about attitude of SHI were considered as having “good attitude” towards SHI, else having “poor attitude”.

#### Data collection procedure

Data were collected using structured, pre-tested and self-administrated questionnaires. The questionnaire was adapted from the International Labour Organization (ILO) and previous studies on demand for social health insurance [26–29]. Five data collection facilitators (BSc in health science) working in nongovernmental organizations in Bahir Dar town were employed to collect the data. Data were collected from April 26, 2013 up to May 10, 2013.

### Data quality control

To ensure the quality of the data, pre-test was conducted on 5% of the sample. Corrections of the questionnaire to contextualize and clarify the questions were made based on the feedback received from the pre-test. The principal investigator trained facilitators of the data collection about the objectives of the study and the contents of the questionnaire, procedures how to obtain consent and techniques to assist respondents, and other ethical issues of autonomy and confidentiality of the respondents. The questionnaires were checked manually for any inconsistencies and incompleteness.

### Data management and analysis

Data were first entered using Epi Info version 3.5.1 and transferred to IBM SPSS Statistics 20 software packages for analysis. Descriptive statistics were performed to present the result in the form of tables, figures and text using frequencies and summary statistics such as mean, standard deviation and percentage. Binary Logistic regression was applied to determine the association of independent variables with the outcome variable (Demand for SHI). All variables having a *p* value of less than 0.2 in the binary logistic regression analysis were entered in the multivariable logistic regression model to control the effect of confounding. The unadjusted model was derived from the binary logistic regression model computed for each variable. The multivariable logistic regression model provides an adjusted model by refining the crude effects of multiple variables which were selected from the binary logistic regression model constructed for each individual variable. A Chi-square test was performed to check independence of categorical variables. Hosmer and Lemeshow test for goodness of fit was performed and which showed significance level above 0.05 indicating the model was good. Variables with a *p*-value of less than 0.05 in the multivariable logistic regression model were considered as statistically significant. The degree of association between independent and dependent variables was estimated using odds ratios (OR) and their respective 95% confidence intervals. Crude OR from the binary logistic regression model were used to measure the crude associations between variables and SHI. Finally, adjusted ORs from the adjusted model were used.

## Results

### Socio-demographic characteristics

A total of 557 civil servants were included in the study. Four hundred and eighty eight (response rate of 88%) returned the questionnaire. More than half, 257(52.7%), of the respondents were males and 315(64.5%) were married. The mean age (SD) of respondents was 35.58 ± 9.45. Majority of the respondents, 429(87.9%), attained a higher educational level. About two-third, 323(66.2%), of the respondents were working as professionals or technical employees (Additional file 3).

The mean family size was 3.57 ± 1.97(SD). More than half of the respondents, 276(56.6%), had 1–3 children and 266(54.5%) were financially responsible for ≤ 2 persons. The mean dependency was 2.5 ± 2.3(SD) people. One hundred and forty respondents (28.7%) had less than 6 years of work experience. Most spouses of respondents, 220(69.8%), were employed. One-fourth of the respondents, 128 (26.2%), were earning more than 3500 Ethiopian birr (about 175 US dollars) monthly (Additional file 1).

#### Demand for social health insurance among civil servants

Majority of the respondents, 355(72.7%), described their need to be enrolled in the SHI scheme. More than half, 325 (66.6%), were willing to pay for their enrollment. Three hundred two respondents (61.9, 95%CI: 57.4, 66.2%) were demanding to be enrolled in SHI.

**Fig. 1:**
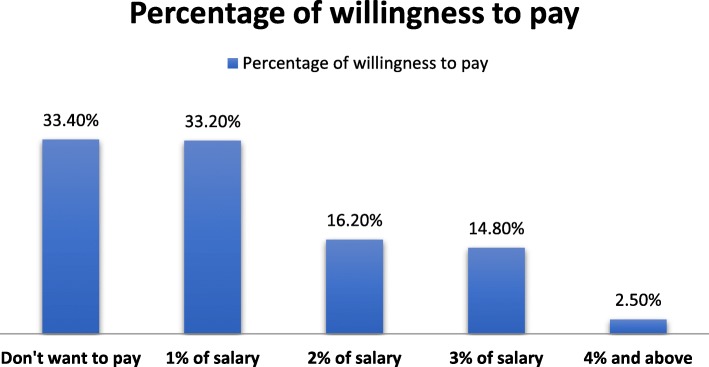
Patterns of willingness to pay for SHI among civil servants in Bahir Dar Town

One third of the respondents, 163 (33.4%) were not willing to pay for SHI. However, another one-third, 162 (33.2) reported they would pay only 1% of their salary for SHI (Fig. 1).

#### Health related characteristics

Regarding the health status of respondents, 343 (70.3%) of respondents reported that they were sick at least once in the last 12 months. From those reported that they were sick at least once in the last 12 months, 123 (35.9%) suffered for an illness which lasted more than a week and 251(73.4%) of the respondents have paid less than 1500 ETB (about 75 USD) for the health service and nearly one-fifth of the respondents, 93(19.1%), were hospitalized. Almost half of the respondents, 235 (48.2%), can access healthcare facility within 15–30 min travel time while 24(4.9%) of participants can reach health facility after travelling at least for an hour. Majority of respondents, 347(71.1%), reported that they don’t trust the quality of health care service they received at the time of their or their families illness (Additional file 3).

From those who demanded SHI, 217 (63.3%) of respondents reported that they were sick at least once in the last 12 months. From those who demand SHI and reported that they were sick at least once in the last 12 months (Table 1).

**Table 1:** Demographic and socio-economic characteristics with bivariate and their respective bivariate logistic regression association with SHI demand

Variables	SHI Demand		Crude OR (95% CI)
	Yes	No	
Age (mean, SD, 35.58 ± 9.45)			
18–29	102(63.4%)	59(36.6%)	0.864(.451–1.65)
30–39	105(60%)	70(40%)	0.750(.395–1.425)
40–49	59(60.2%)	39(39.8%)	0.756(.377–1.516)
50–60	36(66.7%)	18(33.3)	1
Marital status			
Single	91(62.8%)	54(37.2%)	1.264(.556–2.872)
Married	195(61.9%)	120(38.1%)	1.219(.557–2.665)
Separated	16(57.1%)	12(42.9%)	1
Sex			
Male	164(63.8%)	93(36.2%)	1.188(.824–1.713)
Female	138(59.7%)	93(40.3%)	1
Educational status			
Primary education	6(50%)	6(50%)	.595(.189–1.875)
Secondary education	27(57.4%)	20(42.6%)	.803(.436–1.478)
Higher Education	269(62.7%)	160(37.3%)	1
Religion			
Orthodox Christian	288(39%)	450(61%)	*4.000(1.213–13.193)
Muslim	10(28.6%)	25(71.4%)	1.500(.361–6.230)
Others	4(23.5%)	13(76.5%)	1
Family size			
Single Family	70(66.6%)	36((33.4%)	1.027(.559–1.889)
2–3 Family members	77(60.6%)	50(39.4%)	.814(.456–1.453)
4–5 Family members	102(58.6%)	72(41.1%)	.748(.433–1.295)
More than 6 Family members	53(65.4%)	28(34.6%)	1
Total number of dependents			
< 2	164(61.7%)	102(38.3%)	1.206(.650–2.236)
3–5	110(63.6%)	63(36.4%)	1.310(.687–2.496)
6 and above	28(59.6%)	19(40.4%)	1
Total dependent children			
Have no child	108(60%)	72(40%)	.900(.415–1.954)
1-3children	174(63%)	102(37%)	1.024(.480–2.180)
More than 4 children	20(62.5%)	12(37.5%)	1
Employee’s t			
Administrative workers	120(72.7%)	45(27.3%)	**2.066(1.375–3.104)
Professional or Technical Workers	182(56.4%)	141(43.6%)	1
Year of work experience			
< 6 yrs	92(65.7%)	48(343%)	1.323(.799–2.190)
6-12 yrs	63(58.9%)	44(41.1%)	.988(.582–1.679)
12.01-20 yrs	76(62.8%)	45(37.2%)	1.166(.694–1.957)
> 20 yrs	71(59.2%)	49(40.8%)	1
Total monthly family income			
525–1500 ETB	65(64.4%)	36(35.6%)	1.032(.283–3.764)
1501–2500 ETB	84(67.7%)	40(32.3%)	1.200(.332–4.337)
2501–3500 ETB	70(56.5%)	54(43.6%)	.741(.206–2.661)
> 3500 ETB	76(59.4%)	52(40.1%)	.835(.233–2.998)
I don’t Know	7(63.6%)	4(36.4%)	1

*P < 0.05; **P < 0.01

#### Personal behavioral factors of civil servants

Two hundred fifty seven (52.7%) of respondents have poor awareness on the objectives, components, benefits, and packages of SHI. Majority, 203(83.2%), perceive SHI can solve their unexpectedly higher health care service related costs. More than three fourth, 378 (77.5%), of participants have poor attitude on health insurance, risk and health care. Vast majority of study participants, 404 (82.8%), have perceived that their current healthcare payment mechanism is not sufficient to cover the full cost of their healthcare need. Most of the participants, 314 (64.3%), did not have trust on the ability of government’s Health Insurance Agency to offer the intended benefit packages (Additional file 1).

#### Factors associated with civil servants demand of SHI

The binary logistic regression analysis revealed religion, job category, awareness on health insurance, attitude towards health insurance, trust on government Health Insurance Agency (HIA), respondents’ self-reported evaluation of their current healthcare payment option, type of occupation, perceived benefits of health insurance, exposure to television, newspapers, and participation in health insurance awareness creation sessions were found to be associated with the demand of social health insurance at a *p*-value of less than less than 0.2 (Tables 1, 2, 3).

**Table 2:** SHI demand and health related characteristics and their respective bivariate association with SHI demand

Variables	Demand for SHI		Crude OR (95% CI)
	Yes	No	
Physical Acc. to Health care			
< 15 min	85(62%)	52(38%)	0.8170(.327–2.043)
15-30 min	150(63.8%)	85(36.2%)	0.882(0.363–2.147)
30 m-1 h.	51(55.4%)	41(44.6%)	0.622(0.242–1.597)
> 1 h.	16(66.7%)	8(33.3%)	1
Health status in the last 12 months			
Sick	217(63.3%)	126(36.7%)	1.216(0.818–1.808)
Not sick	85(58.6%)	60(41.4%)	1
Duration of illness in fa.			
0 (not sick)	85(58.6%)	60(41.4%)	0.876(0.536–1.432)
< 3 days	70(66%)	36(34%)	1.202(.0699–2.068)
1 week	71(62.3%)	43(37.7%)	1.021(0.604–1.726)
> 1 week	76(61.8%)	47(38.2%)	1
Perceived satisfaction on the quality of healthcare			
Unsatisfied	212(61.1%)	135(38.9%)	0.890(.593–1.335)
Satisfied	90(63.8%)	51(36.2)

**Table 3:** Personal behavioral factors on SHI demand among civil servants in Bahir Dar town bivariate and their respective bivariate logistic regression association with SHI demand

Variables	SHI Demand		Crude OR (95% CI)
	Yes	No	
Awareness			
Good Awareness	168(72.7%)	63(27.3%)	2.448(1.675–3.576)**
Poor Awareness	134(52.1%)	123(47.9%)	1
Attitudes towards SHI			
Good attitude	83(75.5%)	27(24.5%)	2.232(1.381–3.606)**
Poor attitude	219(57.9%)	159(42.1%)	1
Trust on gov’t HIA			
Yes	137(78.7%)	37(21.3%)	3.344(2.185–5.116)**
No	165(52.5%)	149(47.5%)	1
Perceived benefit of SHI			
Helpful	149(73.4%)	54(26.6%)	1
Not helpful	23(56.1%)	18(43.9%)	0.463(0.232–0.924)*
Evaluation of current pay. System			
Sufficient to cover all cost	60(71.4%)	24(28.6%)	1
Not sufficient to cover	242(59.9%)	162(40.1%)	0.598(0.3580.999)*
Television ownership/exposure			
Yes	96(72.8%)	36(27.2%)	1.942(1.254–3.006)**
No	206(57.9%)	150(42.1%)	1
Radio ownership/exposure			
Yes	75(68.8%)	34(21.2%)	1.477(0.938–2.372)
No	227(59.9%)	152(40.1%)	1
Newspaper ownership/exposure			
Yes	52(86.7%)	18(13.3%)	1.941(1.097–3.4350)*
No	250(59.8%)	168(40.2%)	1
Participation in awareness Creation sessions			
Yes	44(77.2%)	13(22.8%)	2.270(1.178–4.399)*
No	258(59.9%)	173(40.1%)	1

*P <0.05; **P < 0.01

However, in the multivariable logistic regression model, only awareness on health insurance, and trust on government HIA were significantly associated with the demand for SHI. Respondents having good awareness on health insurance were 4.39 times more likely to demand SHI than those who had poor awareness. In addition, respondents having trust on the government health insurance agency were 3.0 times more likely to demand SHI than those who did not trust it (Table 4). The goodness of fit of the model was checked using the Hosmer-Lemshow goodness of fit resulting in a *p*-value of 0.83, which indicates the model was good.

**Table 4:** Multivariable logistic regression analysis of factors associated with demand for SHI among civil servants in Bahir Dar town Administration, Northwest Ethiopia (*n* = 488)

Variables	SHI Demand		Crude OR (95% CI)	Adjusted OR (95% CI)
	Yes	No		
Awareness				
Good Awareness	168	63	2.45(1.68–3.56)***	4.39(1.82–12.89)*
Poor Awareness	134	123	1	1
Attitudes towards SHI				
Good attitude	83	27	2.232(1.38–3.61)***	
Poor attitude	219	159	1	
Trust on government HIA				
Yes	137	37	3.344(2.19–5.12)***	3. 0(1.57–5.72)***
No	165	149	1	1
Perceived benefit of SHI				
Helpful	149	54	1	
Not helpful	23	18	0.463(0.232–0.924)*	
Current payment				
Sufficient to cover all cost	60	24	1	
Not sufficient to cover	242	162	0.598(0.36–0.999)*	
Employment				
Administrative worker	120	45	2.066(1.38–3.10)***	
Professional/Technical	182	141	1	
Religion				
Orthodox Christian	288	162	1	
Muslim	10	15	0.375(0.165–0.85)*	
Others	4	9	0.250(0.08–0.83)*	
Television				
Yes	96	36	1.942(1.25–3.01)***	
No	206	150	1	
Radio				
Yes	75	34	1.477(0.94–2.37)	
No	227	152	1	
News paper				
Yes	52	18	1.941(1.09–3.4)*	
No	250	168	1	
Participation in awareness Creation sessions				
Yes	44	13	2.270(1.18–4.4)*	
No	258	173	1	

*p = 0.05–0.01, ***p < =0.01

Hosmer-Lemshow goodness of test for model X^2^ = 4.315, degree of freedom = 8, p-value = 0.83

## Discussion

This study aimed at assessing the demand for social health insurance and associated factors among civil servants living in Bahir Dar city, Ethiopia. The study revealed that two-third of the respondents are willing to pay for their enrollment and 62% demand for SHI. This finding is slightly lower than a study reported in South Ethiopia, Wolaita Sodo (74%) [30] and Southwest Ethiopia, Jimma (84%) [31]. The difference is possibly due to the difference in their awareness. In the current study less than a third of the respondents were aware of the health insurance while in the Wolaita Sodo study about 45% have ever heard of social health insurance [30]. A qualitative study from Addis Ababa reported that there is little knowledge regarding the concept and elements of health insurance [32]. However, it is higher compared to a study conducted in Nigeria [33]. Possibly, this might be due to the study participants’ previous exposure to insurance scheme and their perceived benefit from it. For example 52% of the participant in the Nigerian study demanded to be enrolled in insurance scheme. However, only 0.3% believed that they are benefited from the insurance [33]. Contrary to this, none of the participant of our study had previous health insurance exposure other than a positive hope for receiving a health insurance service. In addition, the ever increasing economic inflation coupled with the rising cost of healthcare service might have made the respondents to look for a savior health insurance.

The current study shows that awareness about social health insurance and having trust on the government’s health insurance agency were found to be associated with the demand for social health insurance. In the literature, awareness about health insurance has been found to be an important factor that influence the demand for health insurance. Good awareness increases the probability of demanding health insurance as well as the willingness to pay for it. Related to this, a study from South Ethiopia indicated that willingness to pay for SHI was more likely among those who have heard about it [30]. Similarly, a study from Nigeria reported that civil servants demand for health insurance was associated with their awareness [33]. In addition, there was a statistically significant difference in SHI demand between respondents who trust the government Health Insurance Agency and those who do not. In the present study, two-third of the respondents did not trust the government’s HIA. Those respondents who trust the governmental HIA demand SHI 3.3 times higher than their counterparts. Similar to this, trust was reported to be as one of the key determinants for a viable health insurance scheme in sub-Saharan Africa [34]. These associations may suggest there is a causal association between demand for SHI and awareness of SHI as well as having trust for the government’s HIA. However, it needs to be verified with further studies.

While there is no clear evidence the higher demand of insurance by one religious group over the other, an empirical analysis on the individual religiosity and preference for social insurance outlined that individuals who are religious will prefer lower levels of social insurance provision than will individuals who are secular, and countries that are more religious on average will have lower levels of welfare state spending [35], However, our multiple logistic regression analysis did not show religion as a predictor variable. Contrary to this, a study from Malaysia, Ghana and India showed religion and race influence individual decision to be enrolled and remain in the insurance schemes [6, 15, 25, 36]. Moreover, a recent systematic review reported factors such as attitude, increase in family size, education level and income were consistently correlated with willingness to pay for health insurance [37]. In our study, none of these factors were found to be statistically significant associated with demand for SHI in our study. In our study, more than three-quarter of participants have poor attitude on SHI. This is possibly due to fear of the monthly premiums, lack of trust to the HIA and limited knowledge to the benefit packages as shown in our results and another study from Addis Ababa [32]. Quantitative studies covering wider geographical area as well as qualitative studies are important to understand the factors influencing demand and willingness to pay for SHI, and institutional stability of the agency providing SHI service. Insurance system is a complex system which requires a coordination of multisector stakeholders coordinated efforts. The higher demand of SHI needs to be matched with the supply side readiness from health care institutions, financial sectors and others to fulfil the insurance driven health care seeking behavior.

Though this study was conducted in 2013, considering Ethiopia still struggling to implement SHI system since 2011 and yet the system has not been in place, the findings are current. The ongoing implementation of SHI needs to consider improving the poor awareness and the lack of trust on HIA through advocacy and awareness creation activities. In addition, improving knowledge regarding the importance of paying monthly premiums and the benefits SHI packages via campaigns and social marketing strategies may help facilitate the implementation of SHI.

The study focused on the demand side of the insurance system to be implemented in Ethiopia, to have a complete picture of the situation further research is needed to assess the readiness of health facilities, insurance agency and the general health information system to shoulder the implementation of social health insurance.

## Limitations of the study

The study has limitations. We assessed demand for SHI from participants who have never participated in any form of health insurance system. Because of this, the respondents might have a higher ambition and expectation from a health insurance before knowing the actual importance of having SHI, which might have increased the magnitude of demand for SHI. Some variables were measured using only a single response questions, e.g satisfaction on the quality of the health care, this might compromise the depth of how much the question accurately measured the variable of interest. Hence, the generalizability of these findings should be interpreted considering this limitations. Regarding the generalizability of our study, as the respondents were selected using appropriate sampling methods and the response rate is higher, we do not believe there would be a significant difference between the characteristics of respondents and non-respondents**.** In addition, variables unobserved in the current study might be predictive of SHI demand. This may have an effect on the internal validity of our results. Our analysis did not assess interaction terms which may have an effect in our results. However, we assessed Chi-square test for independence and we did not observe statistical dependence between variables.

## Conclusion

This cross-sectional survey revealed that civil servants’ demand for SHI prior to the implementation of the insurance scheme is encouragingly high. Good awareness about SHI and having trust on the health insurance agency were found to be positively associated with the demand for SHI among civil servants in the study setting.

## Additional files

Additional file 1: Government offices, total number of civil servants and civil servants proportionally allocated in the sample. (DOCX 17 kb)

Additional file 2: Questionaire. (DOCX 23 kb)

Additional file 3: **Table S1.** Demographic and socio-economic characteristics among civil servants in Bahir Dar town (*n* = 488). (DOCX 19 kb)

## Abbreviations

ETB: Ethiopian birr
HIA: Health Insurance Agency
IRB: Institutional Review Board
OOP: Out of pocket payment
SHI: Social Health Insurance
SPSS: Statistical software package for scientific studies
