# Supplementary material for: Civil servants' demand for social health insurance in Northwest Ethiopia
Source: Arch Public Health. 2018 Sep 13;76:48. doi: 10.1186/s13690-018-0297-x (PMC6136157; doi:10.1186/s13690-018-0297-x)
Supplement: Supplementary file 1 — Government offices, total number of civil servants and civil servants proportionally allocated in the sample. (DOCX 17 kb) [file 13690_2018_297_MOESM1_ESM.docx]

Supplementary material 1: Government offices, total number of civil servants and civil servants proportionally allocated in the sample

| Institutions/offices | Total no. of civil servants | Samples drawn proportionally |
| --- | --- | --- |
| Mayer’s office | **40** | **7** |
| Economy /T/D/S | **341** | **59** |
| Spokesperson office | **7** | **1** |
| Finance/Env/Eco/Dev | **70** | **12** |
| Semi-urban court | **10** | **2** |
| Trade and Transport | **92** | **16** |
| Micro /small scale tra/Industry | **67** | **12** |
| Cooperative association | **16** | **3** |
| Agriculture | **59** | **10** |
| Administration and Security | **9** | **2** |
| Communication | **14** | **2** |
| Militia | **18** | **3** |
| Civil service | **54** | **9** |
| Sport | **26** | **4** |
| Education | **28** | **5** |
| Ladies, Children &Youth | **15** | **3** |
| Culture &Tourism | **21** | **4** |
| Labor & social affairs | **12** | **2** |
| Health office | **22** | **4** |
| Public schools | **1439** | **249** |
| Kebeles | **172** | **30** |
| Millennium parks | **7** | **1** |
| Health centers | **373** | **64** |
| HIV secretariat | **7** | **1** |
| Justice | **31** | **5** |
| Revenue | **114** | **20** |
| Municipal water supply | **167** | **29** |
| TOTAL | **3225** | **557** |
| Mean | **119.67** |  |
| SD | **280.15** |  |
