# Supplementary material for: Civil servants' demand for social health insurance in Northwest Ethiopia
Source: Arch Public Health. 2018 Sep 13;76:48. doi: 10.1186/s13690-018-0297-x (PMC6136157; doi:10.1186/s13690-018-0297-x)
Supplement: Supplementary file 2 — Questionaire. (DOCX 23 kb) [file 13690_2018_297_MOESM2_ESM.docx]

**Section I –Demographic and socioeconomic variables**

| No. | Questions | Responses | Remark |
| --- | --- | --- | --- |
| 101 | Age in years | ------------- |  |
| 102 | Sex | 1.Male 2.Female |  |
| 103 | Religion | 1. Orthodox  2. Muslim  3. protestant  4. others(specify)----------- |  |
| 104 | Marital status | 1. Married  2. Single  3. Divorced  4. Widowed  5. Separated |  |
| 105 | Educational level | 1. Uneducated 2. Primary education  3. Secondary education 4. Higher level education |  |
| 106 | How many people live with you (including you)? | Write number…………… |  |
| 107 | How many of your children depend on you for a living? | Write number…………… |  |
| 108 | For how many of other dependents (apart from your children) you are financially responsible? | Write number…………… |  |
| 109 | Specify in which category of employment are you working now? | 1. Administrative  2.Technical /Professional  3. Other specify……… |  |
| 110 | Your work experience? | ________years |  |
| 111 | What is your spouse employment? | 1. Employed in a regular job  2. Self employed  3. Un employed  4. Retired | If not married skip to Q 114 |
| 112 | If your spouse is employed or self-employed, specify which category | 1.Civil/Public Service 2.Construction 3.Manufacturing 4.Financial/Trading 5.Farming 6.Professional 7.Mining 8.Livestock 9.Fishing 10.Office/administration 11.Employed in private/NGO |  |
| 113 | If your spouse if employed, which sector is she/he employed? | 1. Formal/Regular  2.Informal/Temporary/Contract |  |
| 114 | Estimate your total gross monthly house hold income | 1. 525 to 1000ETB  2. 1001 to 1500] ETB  3. 1501 to 2000 ETB  4. 2001 to 2500 ETB  5. 2501 to 3000 ETB  6. 3001-3500 ETB  7. More than 3500 ETB  8. Don’t know |  |
| 115 | What are the main sources of income for your family? / More than one answer is possible/ | 1. Formal employment for a private company /NGO  2. Formal employment in the state and state enterprises  3. Formal employment for a political party or trade union  4. Self-employment including small-scale trading  5. Farming  6. Pensions  7. Remittances from abroad  8. Other formal income sources | More than one answer will be possible |

**Section II Questions on Health and Health Insurance**

| No. | Question | Response | Remark | | |
| --- | --- | --- | --- | --- | --- |
| 201 | What types of Health facilities are accessible to you? (Multiple response is possible) | 1. Private clinic  2. Pharmacy  3. Government health center  4. Government Hospital  5. Mission Hospital  6. Herbalist  7. Others (give details) |  | | |
| 202 | How far is the nearest health facility from your home in time? | 1. Less than 15m  2. 15-30m  3. 30m-1hr.  4. Greater than 1hr. |  | | |
| 203 | During the past twelve months did you or any member of your family fall sick? | 1. Yes 2. No | If No. skips to Q. 211 | | |
| 204 | If your answer is yes for Q 203 How long was the duration of your sickness? | 1.Less than 3 days  2. Up to 7 days  3. More than 7 days  4. Two weeks and more |  | | |
| 205 | If your answer is yes for Q 203, what type of medical facility did you use? multiple response is possible/ | 1. Private Clinic  2.Chemist/Pharmacy  3. Government Health center 4. Government Hospital  5. Mission Hospital  6. Herbalist 7. Others |  | | |
| 206 | If your answer is Yes for Q 203, how much was the approximate cost of the treatment? | 1. Nothing 2.Less than 500 ETB  3. 500 - 1500 ETB  4. 1501 - 3000 ETB  5. 3001 - 5000 ETB  6. More than 5000 ETB |  | | |
| 207 | If yes for Q ----203 Have you been on hospital admission during the past twelve months? | 3. Yes 4. No |  | | |
| 208 | If yes, for Q ----207 for how long were you admitted? | 1. Less than 1 week 2. Between 1 & 2 weeks  3. More than 2 weeks | If no skip to  Q 211 | | |
| 209 | How much did you pay for your stay in hospital? | 1. Nothing 2.Less than 500 ETB  3. 500 - 1500 ETB  4. 1501 - 3000 ETB  5. 3001 - 5000 ETB  6. More than 5000 ETB  7. Don’t know |  | | |
| 210 | Who pays for your medical costs? | 1. Personal 2. Government 3. Both personally & government 4. Others specify if……….. |  | | |
| 211 | Who should pay for health care services | 1. Individuals alone 2.Government  3. Both individuals & government  4. Employers 5. Don’t know |  | | |
| 212 | The government covers 30% of current health care costs from the revenue collected. Do you think this budget is enough to deliver health service properly/sufficiently? | 1. Yes 2. No |  | | |
| 213 | Does your funding system cover all aspects of your medical expenses? | 1. Yes 2. No |  | | |
| 214 | Does your current funding system cover all aspects of your dependents’ medical expenses? | 1. Yes 2. No |  | | |
| 215 | Are you satisfied with your current payment systems? | 1. Yes 2. No |  | | |
| 216 | Are you satisfied with the quality of treatment you obtained at the medical facility you used? | 1.Not at all satisfied 2.Not satisfied  3. Satisfied  4. Very satisfied |  | | |
| III. **Awareness Assessment Questions** | | | | | |
| 301 | Where do you think health care funds come from? /multiple response is possible/ | 1. Personal (out of pocket) 2. Regular government budget 3. Government HIA 4. Private health insurance  5. Employers 6. International funding agencies. 7. I don’t know | |  | |
| 302 | Do you know what health insurance is? | 1. Yes 2. No | | If no skip  Q. 305 | |
| 303 | If your answer is yes for Q 302, please mention types/methods of health insurance. /multiple response is possible/ | 1. Private for profit health insurance  2. Private for non-profit health insurance  3. Community based health insurance  4. Social health insurance | |  | |
| 304 | If your answer is yes for Q 302, Where did you hear about it?/ multiple response is possible/ | 1. Television 2. Radio  3. Newsletters  4. Awareness creation sessions 5. Others if, specify……….. | |  | |
| 305 | Have you heard that the government has planned to begin the social health insurance scheme for civil servants in the near future? | 1. Yes 2. No 3. Don’t know | | If, No skip to  Q. 308 | |
| 306 | If your answer is yes for Q 305, how should we decide to be enrolled in social health insurance? | 1. Should be voluntarily 2.It should be compulsory to all 3.Others, specify………….. | |  | |
| 307 | If yes, what are the services/ benefit packages for members? /multiple response is possible/ | 1. Out patient service  2. Inpatient services  3. Diagnostic services  4. plastic surgery  5. implantation of artificial denture for beauty purpose  6. Pharmaceuticals/drug supply 7. Other services which are not fundamental to stay alive. | |  | |
| 308 | If yes for Q 305, what are the objectives of social health insurance?  / multiple response is possible/ | 1. Improve access to health care by reducing OOP spending 2. Remove/reduce substantial financial burdens of households during illness 3. Improves quality of care by increasing resources for health care facilities 4. Enhancing accountability 5. Mobilizing additional resources for health sectors through a collection of contributions/premium. | |  | |
| 309 | Do social health insurance solves the problems of health service expense? | 1. All 2. Most 3. Some  4. None 5. Don’t know | |  | |
| 310 | How should collections of premium for SHI be allocated/decided/? multiple response is possible/ | 1. Based on the health status of the member  2. Based on the family size of the number 3. Based on the member’s health service preference  4. Based on the salary/income of a member  5. I don’t know | |  | |
| 311 | Do you know the presence of health services which are not covered by social health insurance services not in the social health insurance benefit package? | 1. Yes  2. No | |  | |
| 312 | If yes for Q.311 please select those health care services which are not in the social health insurance benefit package./ multiple response is possible/ | 1. Out patient service 2. Inpatient services  3. Diagnostic services  4. plastic surgery  5. Implantation of artificial denture for beauty purpose 6. Pharmaceuticals/drug supply 7. Other services which are not fundamental to stay alive. | |  | |
| 313 | Who are beneficiaries of social health insurance?  / multiple response is possible/ | 1. Members 2. Spouse  3. All the children of members 4. Children below age of 18 5. Don’t know | |  | |
| **IV. Demand Related Questions** | | | | | |
| 401 | Do you want to enroll to such an insurance agency? | 1. Yes 2. No | |  | |
| 402 | How much percentage of your salary per month will you pay for your membership of the social health insurance? | 1. I don’t want to pay  2.1%of my salary  3. 2% of my salary  4. 3% of my salary  5. 4% & above | |  | |
| 403 | If not for Q. 401, what is your reason? Please mentions your reason in the space provided. |  | |  | |
| 404 | Do you trust the current government’s social health insurance provider agency? | 1. Yes 2. No | |  | |
| **V.** **ATTITUDE QUESTIONS** | | | | | |
| 501 | Please respond to the following statements:  I’m healthy enough that I really don’t need health insurance; | Choose one response for each  1. Strongly agree  2. Agree somewhat 3. Uncertain  4. Disagree 5. Strongly disagree | | |  |
| 502 | I think health insurance is not worth the money it costs; | 1. Strongly agree  2. Agree somewhat 3. Uncertain 4. Disagree  5. Strongly disagree | | |  |
| 503 | I’m more likely to take risks than the average person; | 1. Strongly agree 2. Agree somewhat 3. Uncertain 4. Disagree  5. Strongly disagree | | |  |
| 504 | I can overcome illness without help from a medically trained person. | 1. Strongly agree  2. Agree somewhat 3. Uncertain 4. Disagree  5. Strongly disagree | | |  |
