# Supplementary material for: Civil servants' demand for social health insurance in Northwest Ethiopia
Source: Arch Public Health. 2018 Sep 13;76:48. doi: 10.1186/s13690-018-0297-x (PMC6136157; doi:10.1186/s13690-018-0297-x)
Supplement: Supplementary file 3 — Table S1. Demographic and socio-economic characteristics among civil servants in Bahir Dar town (n = 488). (DOCX 19 kb) [file 13690_2018_297_MOESM3_ESM.docx]

Table S1 Demographic and socio-economic characteristics among civil servants in Bahir Dar town (n=488)

| **variables** | **Frequency** | **Percentage** |
| --- | --- | --- |
| Age (mean, SD, 35.58 ± 9.45) |  |  |
| 18-29 | 161 | 33 |
| 30-39 | 175 | 35.86 |
| 40-49 | 98 | 20.08 |
| 50-60 | 54 | 11.06 |
| Marital status |  |  |
| Single | 145 | 29.71 |
| Married | 315 | 64.55 |
| Separated | 28 | 5.74 |
| Sex |  |  |
| Male | 257 | 52.66 |
| Female | 231 | 47.34 |
| Educational status |  |  |
| Primary education | 12 | 2. 5 |
| Secondary education | 47 | 9.6 |
| Higher Education | 429 | 87.9 |
| Religion  Orthodox Christian | 450 | 92.2 |
| Muslim | 25 | 5.1 |
| Others | 13 | 2.7 |
| Family size |  |  |
| Single Family | 106 | 21.7 |
| 2-3 Family members | 127 | 26.0 |
| 4-5 Family members | 174 | 35.7 |
| More than 6 Family members | 81 | 16.6 |
| Total number of dependents |  |  |
| <2 | 266 | 54.5 |
| 3-5 | 173 | 35.5 |
| 6-8 | 45 | 9.2 |
| >8 | 4 | .8 |
| Total dependent children |  |  |
| Have no child | 180 | 36.9 |
| 1-3children | 276 | 56.6 |
| More than 4 children | 32 | 6.6 |
| Employee’s Job category |  |  |
| Administrative workers | 165 | 33.8 |
| Professional or Technical Workers | 323 | 66.2 |
| Year of work experience |  |  |
| <6yrs | 140 | 28.7 |
| 6-12yrs | 107 | 21.9 |
| 12.01-20yrs | 121 | 24.8 |
| >20yrs | 120 | 24.6 |
| Total monthly family income |  |  |
| 525-1500 ETB | 101 | 20.7 |
| 1501-2500 ETB | 124 | 25.4 |
| 2501-3500 ETB | 124 | 25.4 |
| >3500 ETB | 128 | 26.2 |
| I don't Know | 11 | 2.3 |
| **Physical Acc. to Health care** |  |  |
| <15 min | 137 | 28.1 |
| 15-30min | 235 | 48.2 |
| 30m-1hr. | 92 | 18.9 |
| >1hr. | 24 | 4.9 |
| **Health status in the last 12months** |  |  |
| Sick | 343 | 70.3 |
| Not sick | 145 | 29.7 |
| **Duration of illness in fa.** |  |  |
| <3days | 106 | 30.9 |
| 1week | 114 | 33.2 |
| >1week | 123 | 35.9 |
| **Hospitalization history** |  |  |
| Yes | 93 | 27.1 |
| No | 250 | 72.9 |
| **Health care costs (OOP)** |  |  |
| Not paid | 38 | 11.1 |
| <1500ETB | 251 | 73.4 |
| 1501-5000ETB | 40 | 11.7 |
| >5000ETB | 13 | 3.8 |
| **Perceived satisfaction on the quality of healthcare** |  |  |
| Very unsatisfied | 136 | 27.9 |
| Not satisfied | 211 | 43.2 |
| Satisfied | 134 | 27.5 |
| Very satisfied | 7 | 1.4 |
| **Awareness** |  |  |
| Good Awareness | 231 | 47.3 |
| Poor Awareness | 257 | 52.7 |
| **Attitudes towards SHI** |  |  |
| Good attitude | 110 | 22.5 |
| Poor attitude | 378 | 77.5 |
| **Trust on gov’t HIA** |  |  |
| **Yes** | 174 | 35.7 |
| No | 314 | 64.3 |
| **Perceived benefit of SHI** |  |  |
| Helpful | 203 | 83.2 |
| Not helpful | 41 | 16.8 |
| **Evaluation of current pay. system** |  |  |
| Sufficient to cover all cost | 84 | 17.2 |
| Not sufficient to cover | 404 | 82.8 |
| **Television ownership/exposure** |  |  |
| Yes | 132 | 27.0 |
| No | 356 | 73.0 |
| **Radio ownership/exposure** |  |  |
| Yes | 109 | 22.3 |
| No | 379 | 77.7 |
| **Newspaper ownership/exposure** |  |  |
| Yes | 70 | 14.3 |
| No | 418 | 85.7 |
| **Participation in awareness Creation sessions** |  |  |
| Yes | 57 | 11.7 |
| No | 431 | 88.3 |
| Total | 488 | 100% |
